# Accuracy and Precision of Third‐Generation Tympanic Thermometers With Varying Calibration Intervals: A Multicenter Cross‐Sectional Study

**DOI:** 10.1155/nrp/8453356

**Published:** 2026-03-11

**Authors:** Mattia Morri, Anna Brugnolli, Lea Godino, Tatiana Bolgeo, Marianna Azzolini, Daniele Ciofi, Menada Gardalini, Claudia Grezzani, Katia Bettini, Domenica Gazineo, Marina Maffeo, Cristiana Forni

**Affiliations:** ^1^ Department of Nursing, Technical and Rehabilitation Care, IRCCS Rizzoli Orthopedic Institute, Bologna, Italy; ^2^ Center for Medical Sciences (CISMed), University of Trento, Trento, Italy, unitn.it; ^3^ Medical Genetics Unit, IRCCS Azienda Ospedaliero-Universitaria di Bologna, Bologna, Italy; ^4^ Department of Research and Innovation (DAIRI) - Azienda Ospedaliero- Universitaria SS Antonio e Biagio e Cesare Arrigo, Alessandria, Italy; ^5^ Health Professions Unit, Integrated University Hospital of Verona, Verona, Italy; ^6^ Department of Healthcare Professionals, Meyer Children’s Hospital – IRCCS, Florence, Italy; ^7^ MDA SR Anesthesia Nursing Service, Integrated University Hospital of Verona, Verona, Italy; ^8^ Department of Anesthesia and Intensive Care 2, S. Chiara Hospital, Trento, Italy, apss.tn.it; ^9^ Governo Clinico e Qualità, IRCCS Azienda Ospedaliero-Universitaria di Bologna, Bologna, Italy; ^10^ Pediatric Intensive Unit, Meyer Children’s Hospital – IRCCS, Florence, Italy

## Abstract

**Aim:**

To evaluate intra‐ and interoperator reliability and accuracy of the third‐generation tympanic thermometer (GeniusTM3) and to explore the relationship between measurement error and calibration intervals of the tympanic thermometer.

**Design:**

Open‐label, pragmatic multicenter cross‐sectional study.

**Methods:**

The study was carried out in intensive care units and operating theaters across five hospitals in Northern Italy. Patients of any age admitted to intensive care units or undergoing surgical procedures in operating theaters between December 2023 and October 2024 were enrolled. Two trained nurses independently took the temperature three consecutive times using the same tympanic thermometer. Core temperature was measured using one of the gold standard methods. The measurement error was calculated as the difference between the gold standard measurement and the measurement made with the tympanic temperature.

**Results:**

A total of 550 patients were enrolled. The precision of the tympanic thermometer was shown to be excellent in both intraobserver and interobserver reliability. The tympanic thermometer consistently underestimated body temperature, with a mean measurement error of 0.3°C and a limits of agreement of ±1.3°C. The generalized linear model (GLM) showed calibration interval, recruiting hospital, and the variable defined by their interaction as factors that were independently and statistically significant predictors of accuracy variation.

**Conclusions:**

The third‐generation tympanic thermometer (GeniusTM3) represents a reliable tool for ensuring high accuracy in terms of both intraoperator and interoperator concordance. However, the accuracy of the device has been significantly affected by the timing of the calibration: a calibration interval of more than 6 months results in a significant deterioration in accuracy.

**Summary**

• Tympanic thermometer consistently underestimated body temperature, with a mean measurement error of 0.3°C and a limits of agreement of ±1.3°C.
• Calibration interval is a factor that was independently and statistically significant as a predictor of accuracy variation.
• Maintaining the accuracy of the tympanic thermometer requires proper maintenance and periodic calibration, which is critical to ensure reliable measurements and support appropriate clinical decisions.
• Practical Implications: The high accuracy of the tympanic thermometer and its simplicity of use make it a useful device in daily hospital practice. However, maintaining its accuracy requires proper maintenance and periodic calibration, which is critical to ensure reliable measurements and support appropriate clinical decisions.

## 1. Introduction

Regular or continuous monitoring of body temperature allows for early diagnosis of numerous pathologies [1–6]. Alterations in core body temperature are associated with adverse clinical outcomes and significantly influence therapeutic decision‐making. Therefore, body temperature has become one of the simplest, most cost‐effective, and clinically relevant vital signs in hospitalized patients [7, 8]. Pulmonary artery catheterization is widely regarded as the “gold standard” for core temperature measurement; however, it is an invasive technique with limited applicability in routine clinical practice. Among invasive methods, esophageal and bladder thermometry have also demonstrated high levels of accuracy, particularly within intensive care units and operating theaters [9–11]. In general inpatient wards, body temperature is commonly assessed using noninvasive methods, such as oral and axillary thermometers. In this context, the infrared tympanic thermometer represents a valid alternative due to its rapid measurement capability, minimal patient discomfort, and ease of use [12–14]. Nevertheless, studies have reported inconsistent and often suboptimal levels of accuracy for these devices [15–19]. Several systematic reviews have demonstrated that tympanic thermometers exhibit sensitivity values ranging from 67% to 83%, which some authors deem acceptable for detecting febrile states in clinical practice [11, 12, 18, 20]. A recent systematic review involving 13 cohort studies (632 patients, 105,375 measurements) indicated that peripheral thermometers (including axillary and infrared tympanic thermometers) tend to underestimate core temperature [9]. Studies by Cutuli et al. [9] and Ehlers et al. [15] reported a mean measurement error of 0.44°C and 0.35°C, respectively, in intensive care patients using tympanic thermometers. Niven et al. [11] hypothesized that calibration could enhance the accuracy range of the tympanic thermometer. Hill and Mitchell [21] considered third‐generation infrared tympanic thermometers to be a reliable tool for assessing clinical conditions in patients suggestive of sepsis or COVID‐19 infection. However, current literature on third‐generation tympanic thermometers is limited, and definitive evidence regarding their accuracy and precision is lacking. Therefore, the aim of this study was to evaluate the precision, intra‐ and interoperator reliability, and accuracy of the third‐generation tympanic thermometer (GeniusTM3) by comparing its measurements with those obtained via established reference standards, namely, pulmonary artery catheter, esophageal probe, and bladder probe. A secondary objective was to explore the relationship between measurement error and calibration intervals of the tympanic thermometer, hypothesizing that longer calibration intervals would be associated with reduced measurement accuracy of the device.

## 2. Methods

### 2.1. Study Design

An open‐label, pragmatic multicenter cross‐sectional study was conducted.

### 2.2. Setting

The study was carried out in general intensive care units, cardiac surgery ICUs for adults, pediatric ICUs, and operating theaters across five hospitals in Northern Italy. These centers routinely utilized the third‐generation infrared tympanic thermometer (GeniusTM3 manufactured by Covidien, Mansfield, MA, USA) for measuring body temperature and at least one of the following invasive “gold standard” methods: bladder probe, esophageal probe, or pulmonary artery catheter.

### 2.3. Study Population

Patients of any age admitted to intensive care units or undergoing surgical procedures in operating theaters between December 2023 and October 2024 were enrolled using convenience sampling. Inclusion required that the measurement of body temperature with at least one gold standard method was planned. Excluded from the study were patients with bilateral auricular inflammatory conditions, a lack of gold standard temperature measurement, and a lack of informed consent by patients or legal guardians. No exclusions were made based on diagnosis, reason for hospitalization, or planned surgery.

### 2.4. Measurement Procedures

Core temperature was measured using one of the gold standard methods. Reference “gold standard” body temperature detection was assessed via pulmonary artery catheter, bladder probe, or esophageal probe. The choice of the gold standard was determined by the clinical protocols of the centers involved [10–12, 22]. The third‐generation tympanic thermometer (GeniusTM3) utilizes an infrared sensor capable of detecting body temperature through the tympanic membrane. The measurement procedure was carried out according to the manufacturer’s instructions and had been standardized between the various centers (Figures S1, S2, and S3). Two trained nurses independently took the temperature three consecutive times using the same thermometer within a five‐minute window. Each patient underwent temperature measurement from a single ear, right or left, depending on patient positioning and on the most comfortable side [18, 20, 23]. If the patient used a hearing aid, it was removed at least 5 minutes before the measurement was taken.

The detection of the gold standard temperature and by the tympanic thermometer was carried out independently by two trained nurses. The tympanic temperature was measured three consecutive times using the same thermometer by the nurses responsible for data collection. All measurements performed by the first and second operators were required to be taken within 5 minutes of the reference method measurement and were recorded on a dedicated data collection sheet.

### 2.5. Outcomes

The primary outcomes of the study are the precision and accuracy of the tympanic thermometer measurement.

Precision was defined as the inter‐ and intrameasurement reliability, and it was assessed by calculating the intraclass correlation coefficient (ICC), which allowed for an index of consistency of the measurements performed by the first and second operators (interobserver reliability) as well as the reliability of the three measurements repeated, made by the same operator (intraobserver reliability). The ICC value could range from a minimum to 0 and a maximum to 1: a score lower than 0.4 indicates poor consistency, a score between 0.4 and 0.59 indicates sufficient consistency, a score between 0.6 and 0.74 indicates good consistency, and a score greater than 0.75 indicates excellent consistency [24, 25]. The accuracy of the measurement was evaluated as the ability of the tympanic thermometer to approximate the actual value of body temperature, reporting the measurement error and the limits of agreement (LoA). The measurement error was calculated as the difference between the body temperature detected with the gold standard measurement and the first measurement made with the tympanic temperature by the first operator. In the literature, a thermometer is considered accurate when the average measurement error is between 0.2° and 0.5°. The LoA were calculated from the standard deviation of the measurement error and were deemed acceptable with an amplitude of 1° [12, 26].

### 2.6. Variables

For each tympanic thermometer used, the date of the most recent calibration was recorded. In the case of newly acquired devices, the date of initial use, as provided by the pharmacies of the centers involved, was also recorded. In this way, tympanic thermometers were divided into three groups: thermometers with a most recent calibration within the past 3 months, those calibrated between 3 and 6 months prior, and those with calibration performed more than 6 months earlier. Additional data collected included the age of the patient, distinguishing between pediatric patients (under 18 years) and adult patients (over 18 years and older), as well as sex, the clinical setting in which the measurement took place (distinguishing between operating theaters and intensive care units), and the hospital where the patient was enrolled.

### 2.7. Sample Size

The primary endpoint is the measurement of agreement between the infrared thermometer and the gold standard, to be assessed by the method of Bland and Altman. To determine the sample size, LOA was used as described by Lu et al. [27]. Wanting to detect a minimum mean difference of 0.3°C, with a LoA of ±1°, a 95% LOA confidence level of 0.05, and an alpha error of 0.05, and wanting to achieve a power of at least 0.9, the minimum number of cases to be enrolled for each center was 75 cases.

### 2.8. Statistical Analysis

Dichotomous and categorical data from the enrolled sample such as age, gender, setting, enrolling hospital, reference measurement method used, and thermometer calibration time were reported in terms of absolute and percentage frequency. Repeatability was assessed with ICC: for interreader repeatability, the single measures, and absolute agreement, the two‐way random effects method was used; for intrareader repeatability, the average measures, and absolute agreement, the two‐way random effects method was used.

Bland–Altman analysis was used to assess the agreement of tympanic thermometer measurements with the reference method. Accuracy was reported as measurement error in terms of mean and standard deviation, and the relative 95% LOA was calculated. The impact of various factors such as age, gender, measurement setting, reference measurement method, and hospital and instrument calibration on the measurement error (difference from gold standard and the tympanic temperature measurement) was assessed using the general linear model (GLM) by entering the factors as fixed effects, age as a covariate, and measurement error as a dependent variable. A backward Wald analysis was used to select the best model and avoid confounding or not‐significant factors. For all tests, *p* < 0.05 was considered significant. All statistical analysis was performed using SPSS V.19.0 (IBM Corp., Armonk, NY, USA).

### 2.9. Ethical Considerations

The study was approved by the Ethics Committee of the promoting center, the Ethics Committee of the Area Vasta Centro Emilia, under protocol number 507/2023/Oss/IOR, as well as by the Ethics Committees of all participating centers. Patient enrollment took place only after the study was submitted, and the informed consent was subsequently signed. The study has been registered in the clinical trial database “ClinicalTrials.gov” under the identification number NCT06103604.

## 3. Results

**Figure 1:**
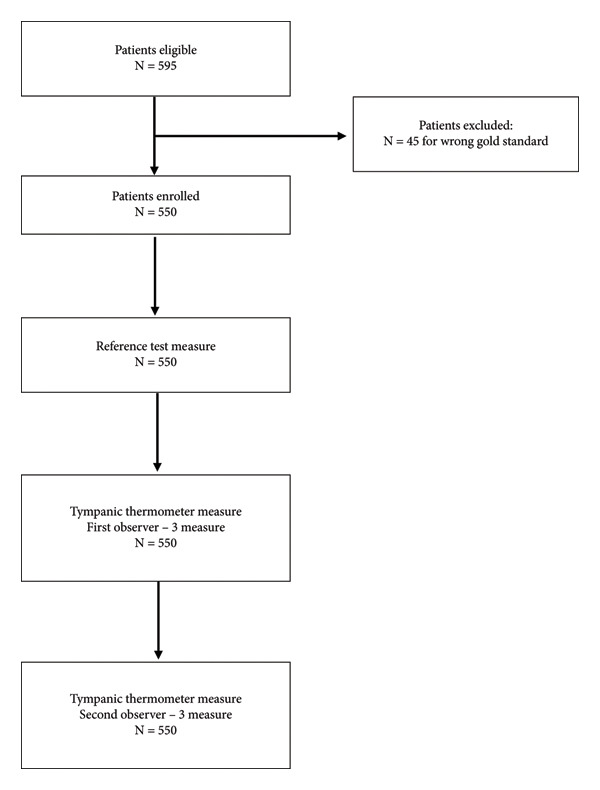
Flow diagram.

A total of 595 patients were deemed eligible for inclusion; 45 were excluded due to a lack of the adequate “gold standard” temperature measurement. The flow of patients is represented in Figure 1. A total of 550 patients were enrolled, with individual hospital recruitment ranging from 52 to 235 patients. The characteristics of the sample are summarized in Table 1. The mean age of patients was 47.7 years (SD 26.0), and 48% were female.

**Table 1:** Baseline characteristics of the sample and univariate analysis for the measurement error.

Variables		*N* = 550	Measurement error, mean (sd)	95% CI	*P* value
Age, *n*	Pediatric	103	0.19 (0.49)	0.09–0.29	0.088
	Adult	447	0.32 (0.70)	0.25–0.38	
Clinical setting, *n*	Operating theaters	374	0.22 (0.73)	0.14–0.29	< 0.005
	Intensive care units	176	0.44 (0.48)	0.37–0.51	
Sex, *n*	Male	286	0.32 (0.61)	0.25–0.39	0.256
	Female	264	0.26 (0.72)	0.17–0.34	
Gold standard methods, *n*	Pulmonary artery catheter	56	0.68 (0.46)	0.56–0.80	< 0.005
	Urinary bladder	165	0.26 (0.96)	0.12–0.41	
	Esophageal probe	329	0.24 (0.47)	0.19‐0‐29	
Calibration, *n*	< 3 months	296	0.21 (0.76)	0.13–0.30	< 0.005
	4–6 months	139	0.33 (0.55)	0.24–0.42	
	> 6 months	115	0.44 (0.50)	0.35–0.53	
Hospital, *n*	A	80	0.53 (0.55)	0‐41‐0.65	< 0.005
	B	127	0.16 (1.03)	−0.03–0.34	
	C	56	0.68 (0.46)	0.56–0.80	
	D	235	0.13 (0.38)	0.08–0.18	
	E	52	0.54 (0.52)	0.40–0.69	

The precision of the tympanic thermometer was shown to be excellent in both intraobserver and interobserver reliability. In the latter case, the level of agreement was similar across the first, second, and third measurements made (Table 2).

**Table 2:** Intra‐ and interreader repeatability for the infrared thermometer.

	ICC	95% CI		*P* value
		Lower limit	Upper limit	
Intrareader for the first reader	0.968	0.963	0.973	< 0.005
Intrareader for the second reader	0.978	0.975	0.981	< 0.005
Interreader for the first measure	0.845	0.814	0.870	< 0.005
Interreader for the second measure	0.857	0.829	0.880	< 0.005
Interreader for the third measure	0.855	0.830	0.876	< 0.005

**Figure 2:**
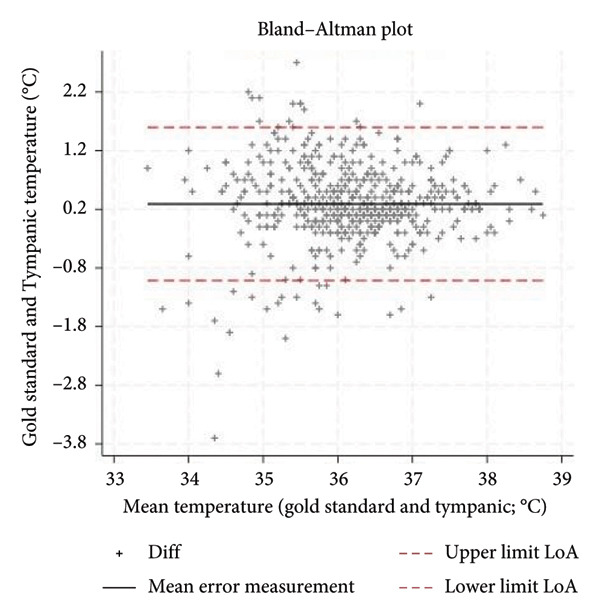
Bland–Altman plot: mean error measurement and LoA.

With respect to accuracy (Bland–Altman analysis), the tympanic thermometer consistently underestimated body temperature, with a mean measurement error of 0.3°C and a LoA of ±1.3°C (Figure 2). The corresponding confidence intervals at 95% were 0.23; 0.35 for the mean measurement error, −1.10; −0.91 for the lower LoA value, 1.49 and 1.68 for the upper value. The recorded temperature measurements ranged from a minimum of 33°C to a maximum of 38.7°C. Measurement error was inversely related to the recency of the device’s calibration: 0.2°C for thermometers calibrated within the previous 3 months, compared to 0.4°C for those with calibration older than 6 months.

Graphical analysis stratified by three calibration intervals demonstrated that the tympanic thermometer with longer calibration intervals tended to more markedly underestimate body temperature compared to the reference method (Figure 3). The three groups defined by the different calibration intervals showed statistically significant differences in accuracy (p value = 0.005) as well as the measurement setting, the recruiting hospital, and the reference method used. The GLM showed calibration interval, recruiting hospital and the variable defined by their interaction as factors that were independently and statistically significant predictors of accuracy variation (Table 3). The coefficient values of the model are provided in Figure (S4). The estimated measurement error, derived from the regression model across different calibration intervals, showed a progressive increase in underestimation error, rising from 0.3 (calibration within 3 months) to 0.5 and 0.7 for calibrations between three to 6 months and beyond 6 months, respectively.

Figure 3Bland–Altman plot by tympanic calibration: (a) calibration ≤ 3 months, (b) calibration 4–6 months, and (c) calibration > 6 months.

**Figure:**
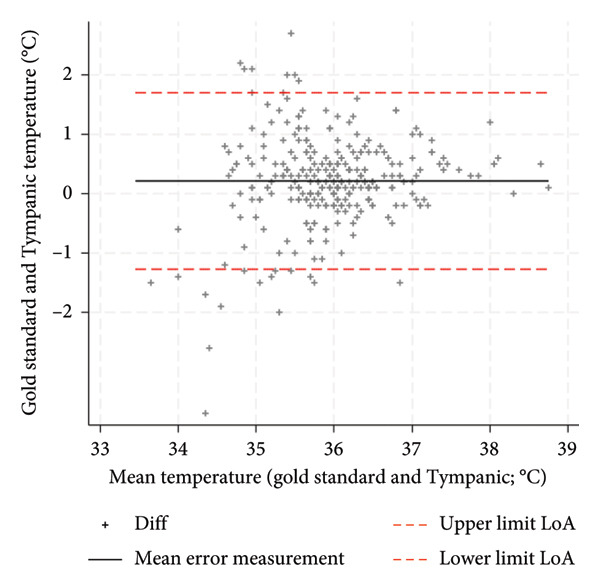
(a)

**Figure:**
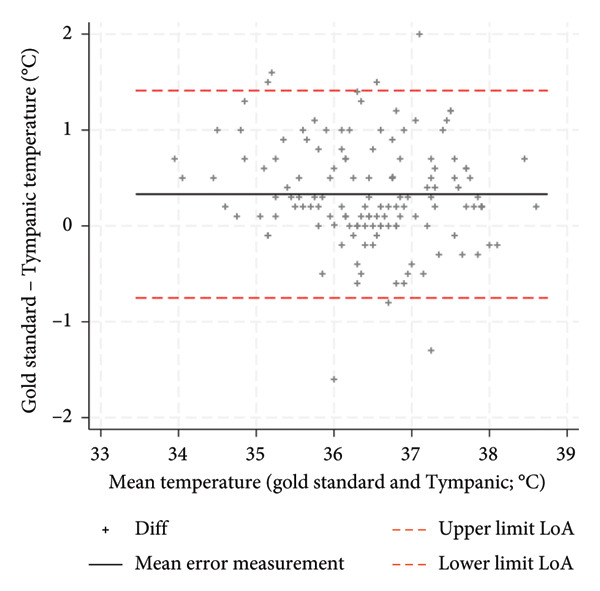
(b)

**Figure:**
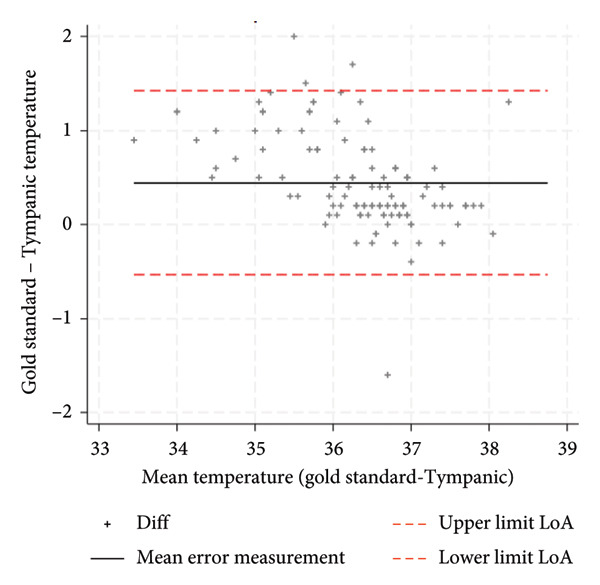
(c)

**Table 3:** General linear model (GLM) between measurement error and variables.

		Estimated mean	95% CI		*P* value	Partial eta squared
			Lower limit	Upper limit		
Hospital	A	0.63	0.46	0.79	< 0.005	0.108
	B	0.16	0.02	0.30		
	C	0.64	0.45	0.83		
	D	0.13	0.02	0.25		
	E	0.59	0.38	0.81		

Calibration	< 3 months	0.29	0.19	0.39	< 0.005	0.032
	3–6 months	0.52	0.38	0.66		
	> 6 months	0.68	0.53	0.82		

Hospital∗calibration					0.029	0.023
Hospital A	< 3 months	0.27	0.08	0.46		
	3–6 months	0.59	0.31	0.88		
	> 6 months	1.02	0.74	1.29		
Hospital B	< 3 months	0.16	0.05	0.27		
Hospital C	< 3 months	0.43	0.07	0.78		
	3–6 months	0.66	0.40	0.91		
	> 6 months	0.85	0.58	1.12		
Hospital D	< 3 months	0.10	0.04	0.24		
	3–6 months	0.13	0.01	0.26		
	> 6 months	0.17	0.03	0.31		
Hospital E	< 3 months	0.50	0.30	0.69		
	3–6 months	0.69	0.35	1.03		

## 4. Discussion

The aim of this study was to evaluate the precision and accuracy of the third‐generation infrared tympanic thermometer. An average measurement error with an underestimation of body temperature of 0.3°C and a LoA of ±1.3°C are in line with the values reported in the literature by some authors on similar measuring devices [21]. Cutuli et al. reported an aggregate measurement error of 0.3°C and a LoA of ±1.4°C for tympanic thermometers, while Niven et al. reported an aggregate measurement error of −0.03°C and a LoA of ±0.9°C. The influence of environmental conditions during temperature measurement and other potential variables that could influence the measurement of temperature needs further investigation to understand whether they may represent the underlying heterogeneity of the results. These variables are less considered in the various studies in the literature. Díaz‐González et al. showed a weak but significant correlation between body temperature measured and ambient temperature and noise levels [28]. An indirect confirmation of this hypothesis can be observed in the fact that, in this study, the accuracy of the tympanic thermometer emerged differently precisely in relation to the recruiting hospital regardless of the setting and the other variables taken into consideration such as age, sex, and the reference method. This factor, in fact, also showed a significant association in multiple regression analysis with respect to the measurement error, potentially reflecting differing environmental conditions across measurement sites. Calibration of the tympanic thermometer was also found to be a significant factor both individually as an association factor and as an interaction with the recruiting hospital. The estimated measurement error, calculated through the multiple regression model after 3 months of calibration, deteriorated significantly to become no longer acceptable after 6 months. This deterioration related to calibration was more marked in some hospitals but present in all the different structures involved. The meta‐analysis by Niven et al. [11] showed a decline in the accuracy of tympanic thermometers when calibration was not performed, with the measurement error increasing from −0.01°C to −0.24°C [11]. Therefore, in the planning of further studies in this area, it is necessary to take into account these aspects, environmental and calibration factors, to fully understand the true accuracy of the third‐generation tympanic thermometer. From the point of view of clinical practice, however, these results suggest the importance of calibrating the tympanic thermometer in order to make it a more accurate instrument. A calibration within 6 months is absolutely necessary to avoid making the temperature measurement inaccurate with an estimated measurement error of 0.7°C and as also visually confirmed by the Bland–Altman plot (Figure 3). The accuracy of the tympanic thermometer was found to be excellent in both intraoperator and interoperator reliability. Having conducted the study within normal clinical practice and therefore having involved multiple professionals in the collection of measurements within different hospitals, it is possible to hypothesize a consistent external validity of the data that emerged.

The results of the study must be read in the light of some considerations and limitations. First of all, different measurement methods such as the esophageal and bladder probe and the pulmonary artery catheter were taken into consideration as reference methods, considering them as a single variable in the calculation of the measurement error. In the literature, there are studies that show a good accuracy of the measurement of the bladder and esophageal probe compared to the measurement in the pulmonary artery, showing a negligible measurement error and LoA [29]. The systematic review conducted by Cutuli et al. [9] showed that, among the invasive methods, the rectal probe was the least accurate compared to the pulmonary artery catheter. For this reason, it was not considered in the present study [9]. The use of a different reference method was analyzed within the regression model as it was not significant and mitigated the possible distortion effect of this aspect. Second, the evaluation of the thermometer was possible only in those hospital contexts in which an invasive reference method for measuring body temperature was used. This aspect is in line with the studies in the literature in which the study population was commonly hospitalized in critical care in order to have the possibility of using appropriate reference methods [9, 20]. This aspect must be taken into account in the evaluation of the external validity of the results, especially toward ordinary hospitalization contexts. At the same time, a strength of the study is contained in its pragmatic nature and in the possibility of having tested the tympanic thermometer on a large number of patients with different characteristics, in different work and climatic contexts so as to still have a robust result in terms of external validity.

Finally, the nurses responsible for data collection were not blinded to the measurement carried out through the standard method. The professionals involved in the data collection carried out this task within normal clinical practice, and this did not allow the observer’s blindness to be realized. The authors believe that the high inter‐ and intraobserver reliability observed in different study contexts, with the involvement of different professionals, makes a systematic measurement error unlikely.

## 5. Conclusions

The results of this study highlight that the third‐generation tympanic thermometer (GeniusTM3) represents a reliable tool for measuring body temperature, ensuring high accuracy in terms of both intraoperator and interoperator concordance. However, the accuracy of the device has been significantly affected by the timing of the calibration: while a calibration performed within 3 months allows for low and clinically acceptable error values, a calibration interval of more than 6 months results in a significant deterioration in accuracy, with an increase in measurement error to levels unacceptable for clinical practice.

## Disclosure

All authors have read and approved the final version of the manuscript.

## Conflicts of Interest

The authors declare no conflicts of interest.

## Author Contributions

Ma.Mo.: analysis and interpretation of the data and drafting of the manuscript. C.F. and A.B.: conception and design, analysis and interpretation of the data, and editing the manuscript. C.G., K.B., G.D., Ma.Ma., L.G., T.B., M.A., D.C., and M.G.: conception and design, acquisition of the data, and editing the manuscript.

## Funding

The authors received no specific funding for this study. Open access funding provided by BIBLIOSAN.

## Supporting Information

Additional supporting information can be found online in the Supporting Information section.

The supporting information provide information on the technical specifications, user and maintenance manual of the tympanic thermometers. Additional data related to the statistical analysis performed are also included.

## Supporting information


**Supporting Information 1** Figure S1: Genius TM3, operating manual.

**Supporting Information 2** Figure S2: Genius TM3, instructions for use.

**Supporting Information 3** Figure S3: Genius TM3, advice for calibration.

**Supporting Information 4** Figure S4: Table of coefficients for the GLM.

## Data Availability

The data that support the findings of this study are available from the corresponding author upon reasonable request.
