# Supplementary figures and images for: Accuracy and Precision of Third‐Generation Tympanic Thermometers With Varying Calibration Intervals: A Multicenter Cross‐Sectional Study
Source: Nurs Res Pract. 2026 Mar 11;2026:8453356. doi: 10.1155/nrp/8453356 (PMC12977293; doi:10.1155/nrp/8453356)

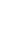

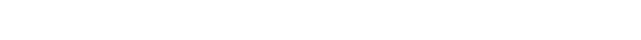

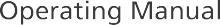

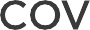

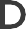

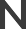

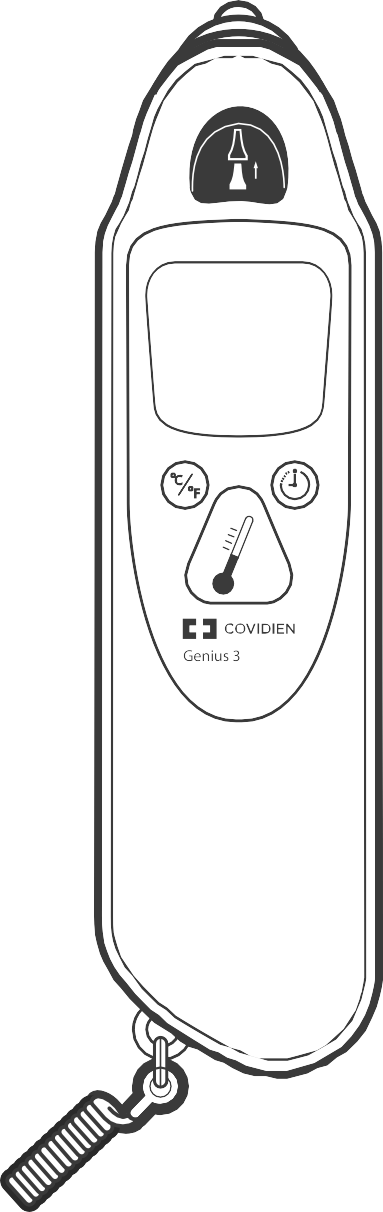


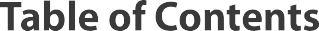


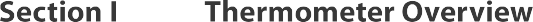

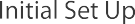

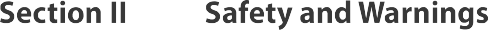

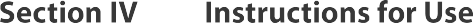

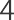

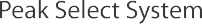

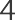

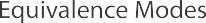

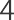

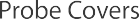

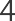

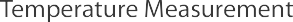

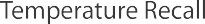

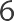

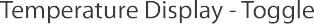

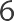

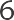

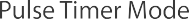

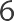

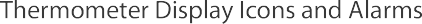

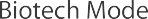

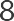

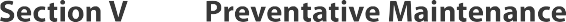

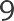

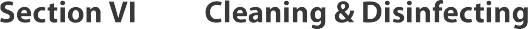

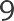

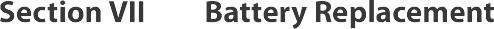

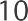

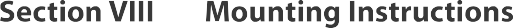

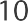

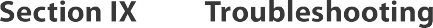

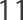

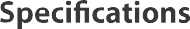

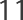

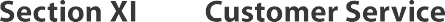

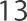

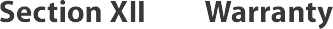

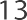

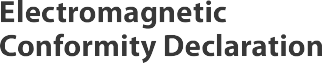

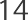


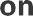

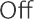

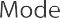


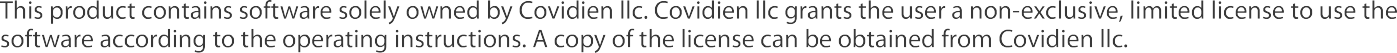

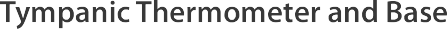


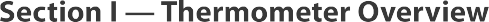


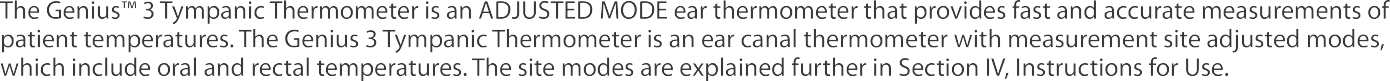

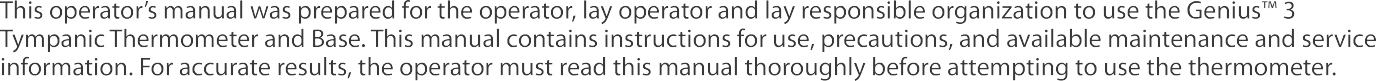

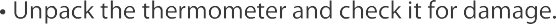

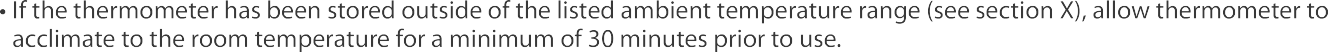

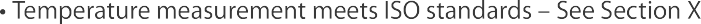

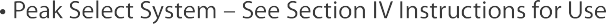

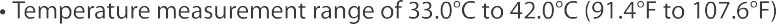

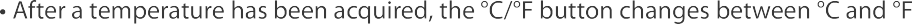

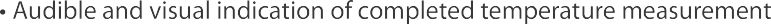

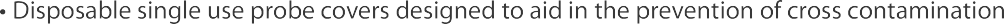

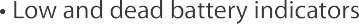

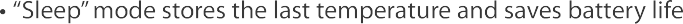

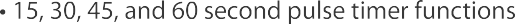

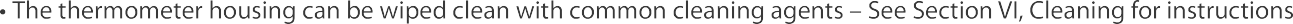

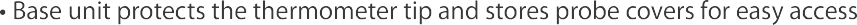

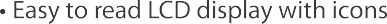

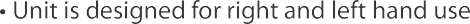

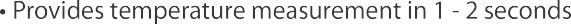

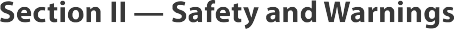

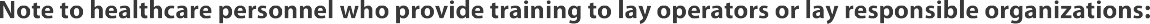

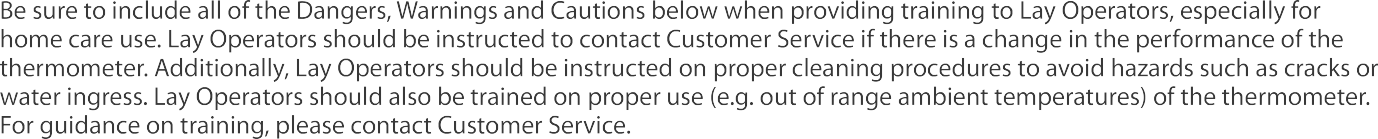

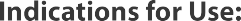

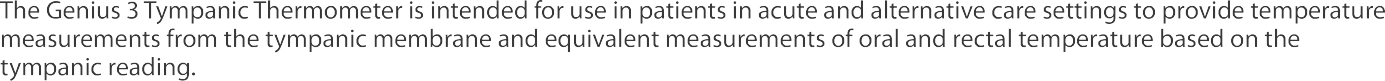

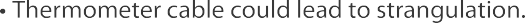

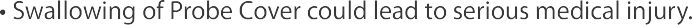


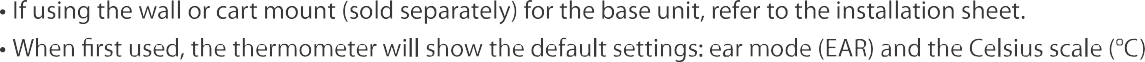

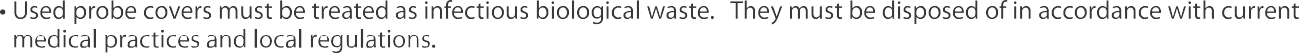


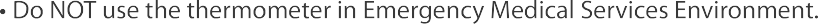

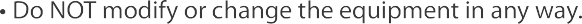

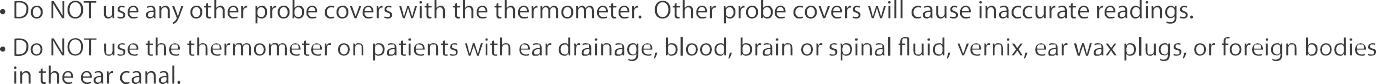

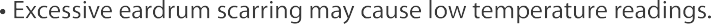

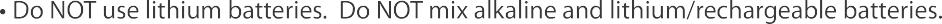

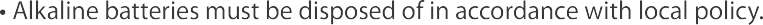

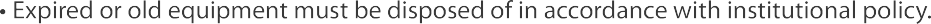

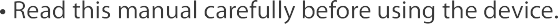

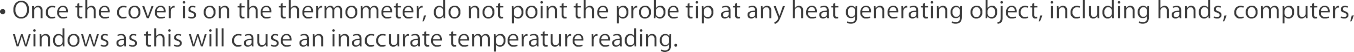

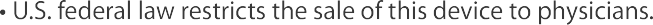

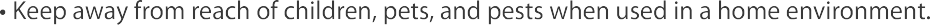

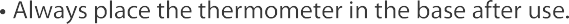

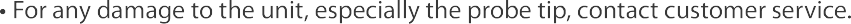

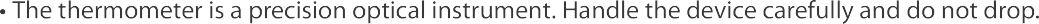

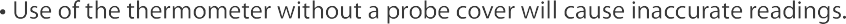

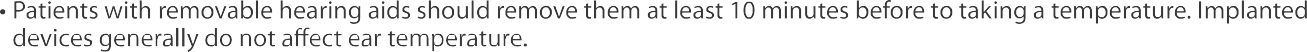

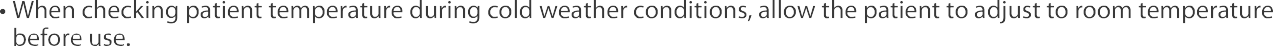

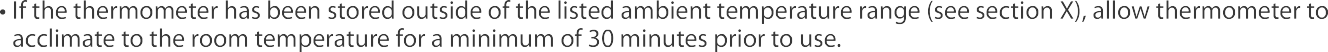

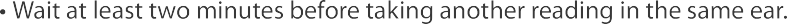


|  | | |
| --- | --- | --- |
|  | | |
|  |  |  |
|  |  |  |
|  |  |  |
|  |  |

|  | | | |
| --- | --- | --- | --- |
|  | | | |
|  |  |  |  |
|  |  |  |  |
|  |  |  |  |
|  |  |  |  |
|  |  |  |  |
|  |  |  |  |
|  |  |  |  |
|  | | | |

|  | | | |
| --- | --- | --- | --- |
|  | | | |
|  |  |  |  |
|  |  |  |  |
|  | | | |
|  | | | |

|  | | | |
| --- | --- | --- | --- |
|  | | | |
|  |  |  |  |
|  |  |  |  |
|  |  |  |  |
|  |  |  |  |
|  |  |  |  |
|  |  |  |  |
|  |  |  |  |
|  |  |  |  |
|  |  |  |  |
|  |  |  |  |
|  |  |  |  |
|  |  |  |  |
|  |  |  |  |
|  |  |  |  |
|  |  |  |  |
|  |  |  |  |
|  | | | |
|  | | | |
|  | | | |
|  | | | |

Supplement: Supplementary file 1 — Supporting Information 1 Figure S1: Genius TM3, operating manual. [file NRP-2026-8453356-s003.docx]
