# Supplementary material for: Accuracy and Precision of Third‐Generation Tympanic Thermometers With Varying Calibration Intervals: A Multicenter Cross‐Sectional Study
Source: Nurs Res Pract. 2026 Mar 11;2026:8453356. doi: 10.1155/nrp/8453356 (PMC12977293; doi:10.1155/nrp/8453356)
Supplement: Supplementary file 2 — Supporting Information 2 Figure S2: Genius TM3, instructions for use. [file NRP-2026-8453356-s002.docx]

Taking a Temperature


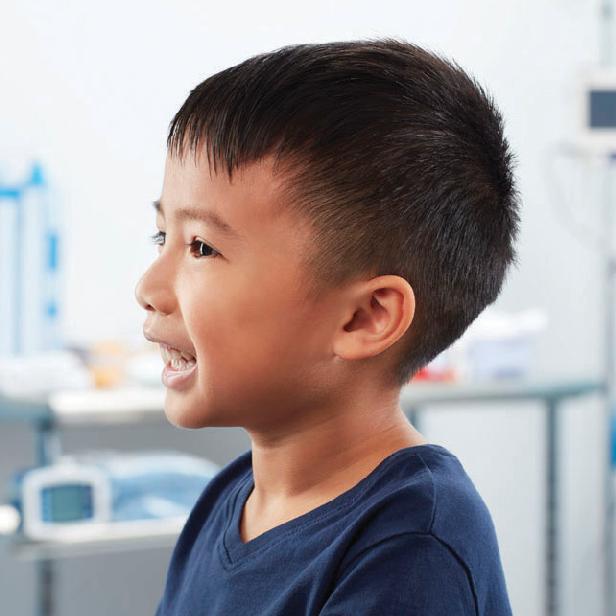

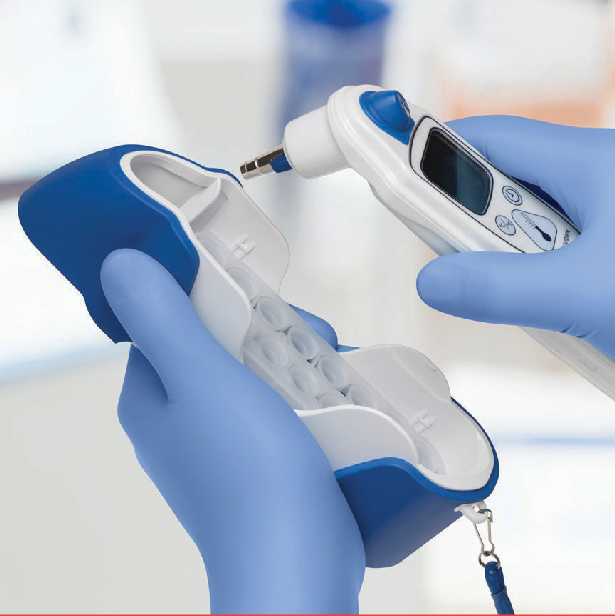

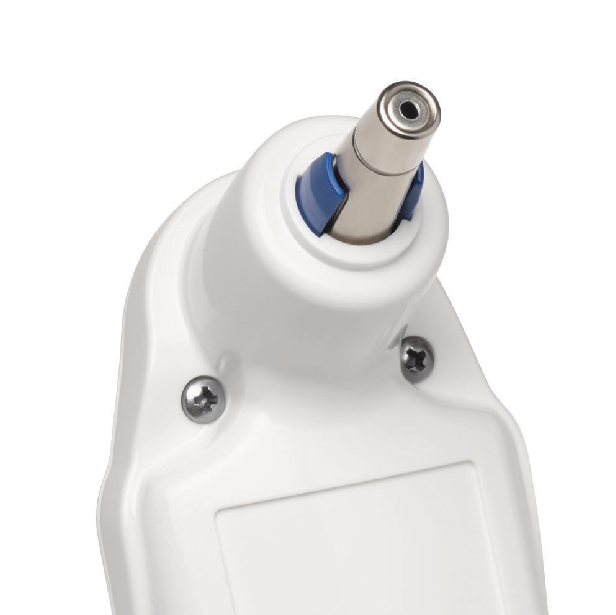


Inspect the patient’s ear anatomy for proper probe

placement. (No blood or fluid should be present.)

Remove the thermometer from the base. Inspect the probe lens. It should be shiny and free of cracks

or debris without a probe cover attached. (Clean lens with a dry lint-free swab or lens wipe if needed.)


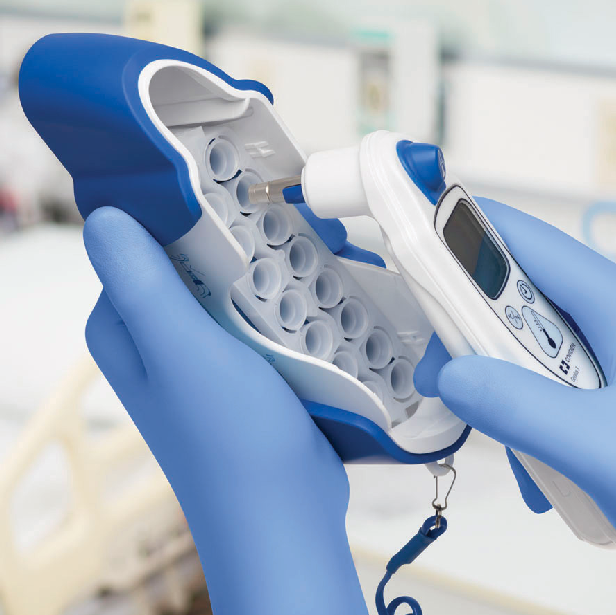

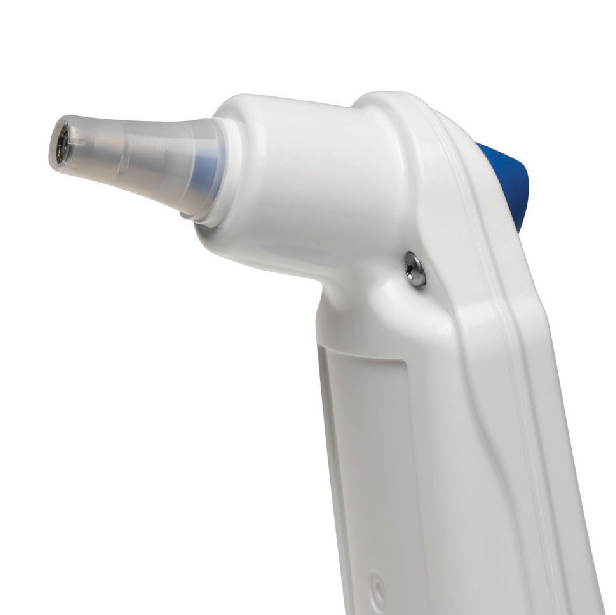

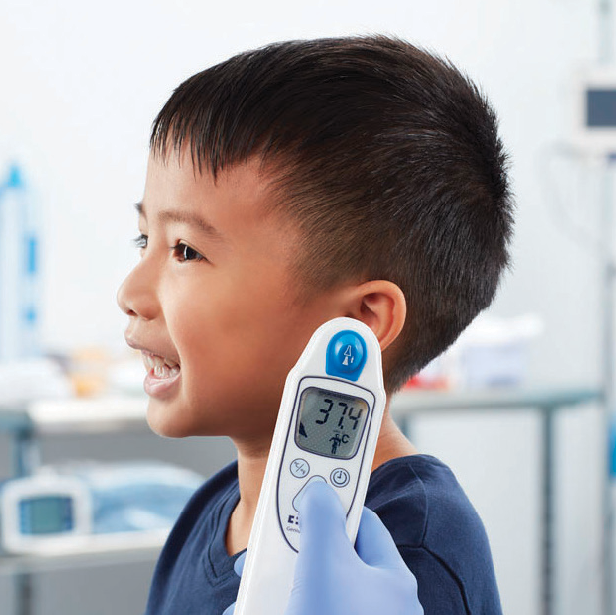

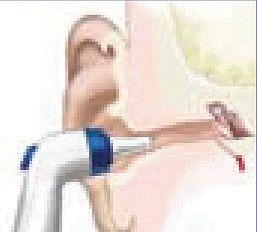


Attach a probe cover by aligning the silver tip with a probe cover and push straight down until you hear a snap. Inspect the membrane on the end of the probe cover. If a hole or tear is visible, discard and attach a new probe cover.

Ensure the probe cover is securely seated on the probe. The silver probe should not be showing. A probe cover MUST be used.

Insert the tapered probe tip. Align the probe at the entrance of the patient’s ear canal. Immediately press the large triangular scan button located in the center of the handle. A triple beep will sound when the scan is completed.


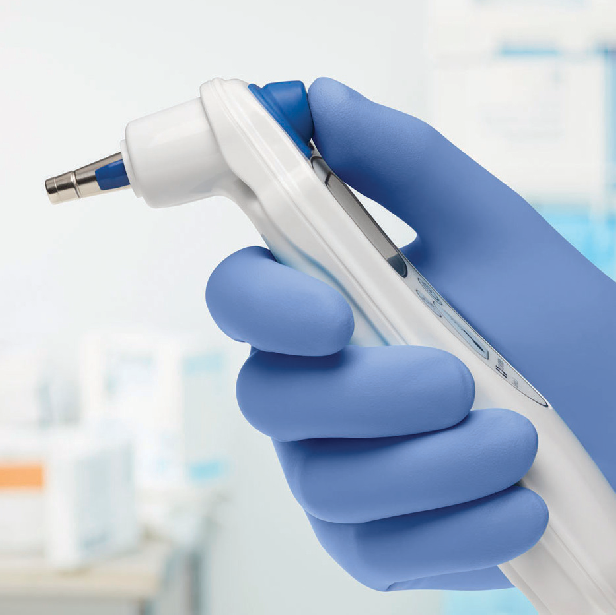

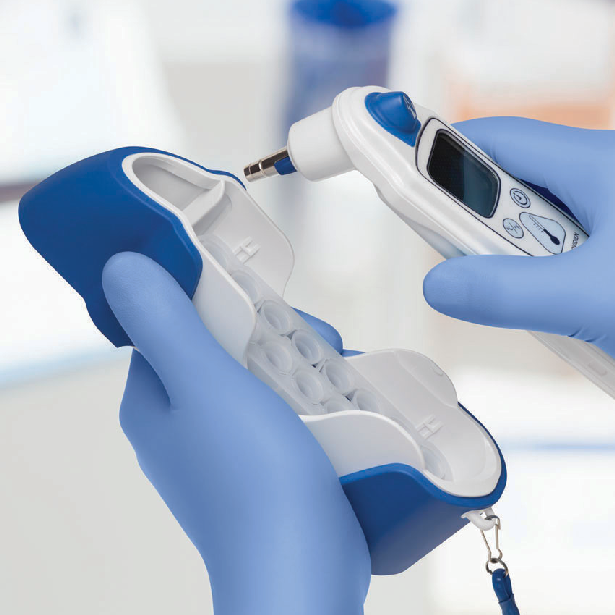

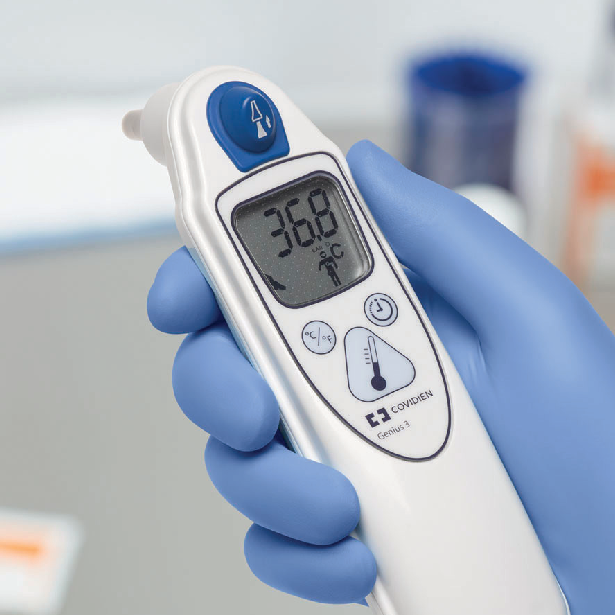


Press the eject button to dispose of the probe cover in a waste receptacle.

**Optional Functions**

Return the thermometer to the base unit for storage. The LCD display will go into “sleep” mode after 10 seconds of inactivity.

To recall the last temperature, press and release the scan button before loading another probe cover. Loading a new probe cover will erase the last temperature taken from

the memory.

**Use ONLY the approved cleaning methods outlined in the Operating Manual.**


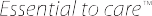

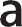

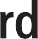

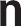

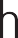


**Genius™ 3** Tympanic Thermometer

**Instructions for use**

To change the temperature display, press and hold the Cº/Fº button for 2 seconds when the LCD screen is active.


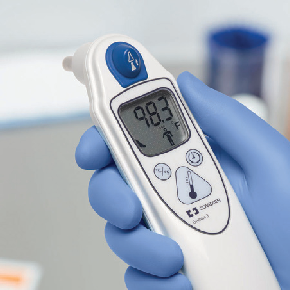

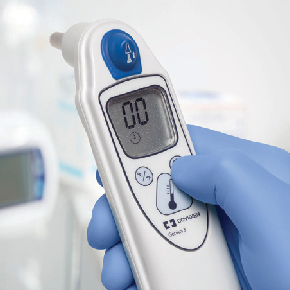


To use the 60-second pulse timer, press and hold the clock icon button for 2 seconds to activate.

Press and release the clock icon button once to


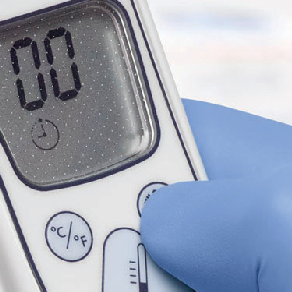


begin. (Beeps will be heard at 15-second intervals.) The thermometer will automatically shut off at 60 seconds. If finished before a full 60 seconds, press and

release the clock icon button again to deactivate.
