# Supplementary material for: Accuracy and Precision of Third‐Generation Tympanic Thermometers With Varying Calibration Intervals: A Multicenter Cross‐Sectional Study
Source: Nurs Res Pract. 2026 Mar 11;2026:8453356. doi: 10.1155/nrp/8453356 (PMC12977293; doi:10.1155/nrp/8453356)
Supplement: Supplementary file 3 — Supporting Information 3 Figure S3: Genius TM3, advice for calibration. [file NRP-2026-8453356-s001.docx]

**Cardinal Health Italy 509 S.r.l.,**


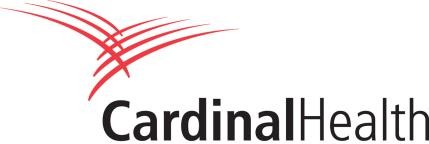
con socio unico

Sede operativa: Via Ostiense, 131/L, Scala C – V Piano C/O Regus, CAP: 00154 Roma, Italia

Tel.: +39 06 833 61 889

Fax: +39 06 833 61 890

30 ottobre 2019

# URGENTE: AVVISO DI SICUREZZA (CORREZIONE)

## Specifici codici prodotto e numeri di serie dei Termometri Genius 2 e Genius 3 ID EVENTO FABBRICANTE: Event-2019-02412

**Alla c.a. di: Direttore Sanitario e/o Responsabile della Vigilanza dei Dispositivi Medici**

Gentile Cliente,

Lo scopo di questa lettera è quello di informare i nostri clienti di un problema relativo ai termometri timpanici Genius 2 e Genius 3.

| **Codice**  **Prodotto** | **Descrizione** | **Prodotto interessato dall’azione** |
| --- | --- | --- |
| 303000 | Termometro timpanico Genius 2  (discontinuato) | Tutti i prodotti fabbricati dopo il 1 Ottobre 2016;  numeri di serie ≥ N16598087 |
| 303013 | Termometro timpanico Genius 3 | Tutti i prodotti fabbricati dopo il 4 Dicembre 2017;  numeri di serie ≥ N17700101 |

**Descrizione dell'avviso:**

La frequenza di calibrazione per il termometro timpanico Genius, come indicato nel manuale d’uso, potrebbe non garantire che i termometri rimangano sempre all'interno dei limiti di accuratezza dichiarati (± 0,2 °C per i termometri Genius 2 e ± 0,3°C per i termometri Genius 3). I valori misurati aumentano nel tempo, il che significa che i termometri potrebbero superare il limite maggiore di accuratezza dichiarata di +0,2°C per il Genius 2 e +0,3°C per il Genius 3. I potenziali danni per il paziente comprendono diagnosi errate e/o ritardo nel trattamento; tuttavia, la probabilità che si verifichino tali circostanze è bassa. Non sono state riportate segnalazioni di lesioni gravi o danni a carico di pazienti.

Cardinal Health sta aggiornando il manuale d’uso per raccomandare la calibrazione dei termometri ad una maggiore frequenza, come indicato nella tabella seguente. Copie dei manuali aggiornati sono disponibili ai seguenti indirizzi:

AVVISO DI SICUREZZA URGENTE (CORREZIONE) – ID EVENTO: Event-2019-02412

**Cardinal Health Italy 509 S.r.l.** con socio unico. Cap. Soc. 10.000,00 euro i.v. REA 2072508. N. Iscrizione Registro delle Imprese Milano

CF/ P.IVA 09158150962 PEC: [cardinalhealthitaly509@legalmail.it](mailto:cardinalhealthitaly509@legalmail.it) Sede legale: Corso Vercelli 40, 20145 Milano, Italia

- Termometro timpanico Genius 2 [https://www.cardinalhealth.com/content/dam/corp/web/documents/Manual/cardinal-health-genius-2-](https://www.cardinalhealth.com/content/dam/corp/web/documents/Manual/cardinal-health-genius-2-operating-manual.pdf) [operating-manual.pdf](https://www.cardinalhealth.com/content/dam/corp/web/documents/Manual/cardinal-health-genius-2-operating-manual.pdf)
- Termometro timpanico Genius 3 [https://www.cardinalhealth.com/content/dam/corp/products/professional-products/ous-patient-](https://www.cardinalhealth.com/content/dam/corp/products/professional-products/ous-patient-recovery/documents/cardinal-health-genius-3-user-manual-2.pdf) [recovery/documents/cardinal-health-genius-3-user-manual-2.pdf](https://www.cardinalhealth.com/content/dam/corp/products/professional-products/ous-patient-recovery/documents/cardinal-health-genius-3-user-manual-2.pdf)

Il Checker/Calibratore Genius (codici prodotto 303096 e 303097) dovrà ricevere un aggiornamento del software per restringere i limiti di tolleranza di calibrazione. Questo garantirà che i termometri Genius rimangano entro i limiti di accuratezza nel tempo che intercorre tra le calibrazioni previste.

| **Modello termometro** | **Attuale frequenza di calibrazione** | **Frequenza di calibrazione aggiornata** |
| --- | --- | --- |
| Genius 2 e Genius 3 | Una per anno (52 settimane) | 25 Settimane dalla data di fabbricazione ed ogni 25 settimane a seguire |

La data di fabbricazione ed il numero di serie possono essere chiaramente individuati sull’etichetta del numero di

serie, come mostrato a seguire:


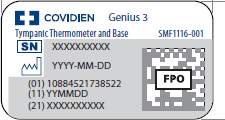
Numero di Serie


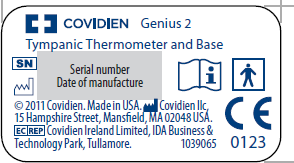


Data di fabbricazione

**Azioni da intraprendere:**

1. Controllare immediatamente i propri inventari per determinare se si è in possesso di unità dei codici prodotto aventi i numeri di serie/data di fabbricazione interessati dalla presente azione.
2. **SE SI DISPONE DI UN CHECKER/CALIBRATORE GENIUS:** calibrare tutti i termometri Genius interessati.

- dopo la calibrazione, contattare il proprio Rappresentante Cardinal Health per pianificare e organizzare l'aggiornamento del software del proprio Checker/Calibratore Genius.
- Una volta che le è stato restituito il Checker/Calibratore Genius aggiornato, ricalibrare tutti i termometri.

**SE NON SI HA ACCESSO AD UN CHECKER/CALIBRATORE GENIUS**: contattare il proprio Rappresentante Cardinal Health.

1. Condividere la presente comunicazione con tutto il personale all’interno della struttura che necessita di essere messo al corrente di questo avviso. Contattare anche qualsiasi altro cliente/struttura/presidio/reparto a cui siano state distribuite o trasferite unità dei prodotti interessati dal presente avviso. A corredo della notifica ai vostri clienti è possibile includere una copia di questa lettera.
2. Leggere, compilare e firmare il Modulo di Conferma Ricezione allegato secondo le istruzioni fornite all’interno del modulo. Restituire il modulo al proprio Rappresentante Cardinal Health o ai contatti indicati all’interno del modulo stesso.

È stato notificato alle Autorità Competenti interessate e all’Ente Notificato che Cardinal Health sta volontariamente intraprendendo questa azione.

Ci scusiamo per eventuali inconvenienti che questo Avviso di Sicurezza potrebbe causare. Per eventuali domande sul presente avviso, si prega di contattare il proprio rappresentante di zona o l’ufficio vendite locale.

**Allegati**

Allegato 1: Modulo di Conferma Ricezione

Cordiali Saluti,


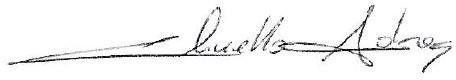


**Andrea Chinello**

**Sales & Marketing Director Italy Medical Solutions - Cardinal Health**

# AVVISO DI SICUREZZA URGENTE (CORREZIONE)

**(ID Evento: 2019-02412)**

# Modulo di Conferma Ricezione

## Termometri Timpanici Genius 2 e Genius 3 - Codici prodotto 303000 e 303013

Cardinal Health sta informando i propri clienti di un problema relativo ai termometri timpanici Genius 2 e Genius 3. La frequenza di calibrazione per i termometri timpanici Genius indicata nel manuale d’suo potrebbe non garantire che i termometri rimangano costantemente all’interno dell’intervallo di accuratezza dichiarato (± 0.2°C per Genius 2 e ± 0.3°C per Genius 3). I valori misurati aumentano nel tempo, il che significa che i termometri potrebbero superare il limite maggiore di accuratezza dichiarata di +0,2°C per il Genius 2 e +0,3°C per il Genius 3.

Questa azione coinvolge tutti i termometri timpanici Genius 2 e Genius 3 oggetto dell’Avviso di Sicurezza

sopramenzionato.

**Attenzione: NON è un richiamo di prodotto**.

I nostri registri indicano che la Vostra Struttura ha ricevuto i dispositivi oggetto dell’Avviso di Sicurezza

sopramenzionato datato 30 ottobre 2019.

Vi preghiamo di restituire il presente modulo, debitamente compilato e firmato, il prima possibile al proprio Rappresentante Cardinal Health o al seguente contatto interno tramite fax o e-mail:

**Cardinal Health Italy 509 S.r.l.**

**c.a. UFFICIO QRA fax: +39 06 83361890**

**e-mail:** [**GMB-QRA-IT@cardinalhealth.com**](mailto:GMB-QRA-IT@cardinalhealth.com)

Indicare le quantità di prodotto identificate che richiedono la correzione oggetto del presente avviso di sicurezza nella tabella sottostante:

| **Codice Prodotto** | **Quantità** |
| --- | --- |
|  |  |
|  |  |

Confermiamo di aver ricevuto e compreso l’Avviso di Sicurezza in oggetto, e di averlo trasmesso a tutti i clienti/strutture/presidi/reparti a cui possano essere stati traferiti i dispositivi interessati da quest’azione.

Confermiamo inoltre (selezionare una delle seguenti opzioni):


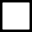
 di disporre di un Checker/Calibratore Genius e di procedere quanto prima alla calibrazione di tutti i termometri Genius interessati. A seguito della calibrazione, contatteremo il Rappresentante Cardinal Health per programmare ed organizzare l’aggiornamento del software del Checker/Calibratore Genius. Una volta che ci sarà restituito il Checker/Calibratore Genius aggiornato, effettueremo una nuova calibrazione su tutti i termometri.


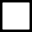
 di non avere accesso ad un Checker/Calibratore Genius. Contatteremo quindi il Rappresentante Cardinal Health.

| **ENTE/STRUTTURA**  **OSPEDALIERA/ DISTRIBUTORE:** | VIA CITTÀ PROV. | | | | |
| --- | --- | --- | --- | --- | --- |
| **NOME E COGNOME:**  (in stampatello) |  | | | | |
| **TITOLO:** |  | | | TEL: |  |
| **DATA:** | / / | **FIRMA*:** |  | | |

** La sua firma ci fornisce conferma della ricezione e comprensione del presente Avviso di Sicurezza.*

VI PREGHIAMO DI INDICARE UN RIFERIMENTO ALL’INTERNO DELLA VOSTRA STRUTTURA PER LE AZIONI IN OGGETTO

AL PRESENTE AVVISO:

NOMINATIVO TELEFONO

E-MAIL

**Si prega cortesemente di restituire tutte le pagine del presente modulo.**
