# Supplementary material for: Accuracy and Precision of Third‐Generation Tympanic Thermometers With Varying Calibration Intervals: A Multicenter Cross‐Sectional Study
Source: Nurs Res Pract. 2026 Mar 11;2026:8453356. doi: 10.1155/nrp/8453356 (PMC12977293; doi:10.1155/nrp/8453356)
Supplement: Supplementary file 4 — Supporting Information 4 Figure S4: Table of coefficients for the GLM. [file NRP-2026-8453356-s004.docx]

***S4. Table of coefficient for the General Linear Model***

*Y= measurement error*

*X= hospital, calibration, hospital*calibration*

| Variable | B | Standard error | t | Sign. | CI 95% | | Partial Eta Square |
| --- | --- | --- | --- | --- | --- | --- | --- |
|  |  |  |  |  | Lower Limit | Upper Limit |  |
| Intercept | ,738 | ,199 | 3,699 | ,000 | ,346 | 1,129 | ,025 |
| Hospital A | ,277 | ,243 | 1,140 | ,255 | -,200 | ,755 | ,002 |
| Hospital B | -,339 | ,114 | -2,971 | ,003 | -,563 | -,115 | ,016 |
| Hospital C | ,110 | ,241 | ,456 | ,649 | -,364 | ,584 | ,000 |
| Hospital D | -,567 | ,186 | -3,054 | ,002 | -,932 | -,202 | ,017 |
| Hospital E | 0^a^ | . | . | . | . | . | . |
| Calibration <3 months | -,243 | ,223 | -1,088 | ,277 | -,681 | ,195 | ,002 |
| Calibration 3-6 months | -,045 | ,099 | -,456 | ,649 | -,240 | ,150 | ,000 |
| Calibration >6 months | 0^a^ | . | . | . | . | . | . |
| Hosptal A; <3months | -,506 | ,280 | -1,806 | ,071 | -1,057 | ,044 | ,006 |
| Hospital A; 3-6months | -,375 | ,223 | -1,681 | ,093 | -,813 | ,063 | ,005 |
| Hospital A; >6 months | 0^a^ | . | . | . | . | . | . |
| Hospital B; <3months | 0^a^ | . |  | . | . | . | . |
| Hospital C; <3months | -,180 | ,317 | -,567 | ,571 | -,803 | ,443 | ,001 |
| Hospital C; 3-6 months | -,146 | ,213 | -,685 | ,493 | -,564 | ,272 | ,001 |
| Hospital C; >6 months | 0^a^ | . | . | . | . | . | . |
| Hospital D; <3 months | ,176 | ,223 | ,792 | ,428 | -,261 | ,613 | ,001 |
| Hopital D; 3-6 months | 0^a^ | . | . | . | . | . | . |
| Hospital D; >6 months | 0^a^ | . | . | . | . | . | . |
| Hospital E; <3 months | 0^a^ | . | . | . | . | . | . |
| Hospital E; 3-6months | 0^a^ | . | . | . | . | . | . |
